# On the Extraction of Teeth

**Published:** 1872-11

**Authors:** John M'Calla


### ARTICLE II.

### *On the Extraction of Teeth.*

BY JOHN M'CALLA, D. D. S.

Read before the Pennsylvania State Dental Society.

The proper treatment of any of the ills which afflict humanity should be the subject of patient and scientific research on the part of every one connected in any way with the healing art. This is no less true of the dental than of the general surgeon. The organs with which he has to deal are most intimately connected with nerves which throb with the keenest agony in suffering, or thrill with the most delightful sensations of pleasure and enjoyment in health.—How skillful, then, should be the hand, and perfect the knowledge of the operator whose field of labor lies so near to the sensorium of the system. We are apt to suppose, in this enlightened age, that a higher point has been attained on the steep declivity of the hill of science, than our predecessors ever reached, and while we admit that rapid strides have been made in this direction, let us not forget the fact, that long before the beginning of the Christian era, the reputation of the Egyptian physicians and surgeons extended throughout the then known world; the monarchs of Persia and other countries for many ages employed them alone. The prevalence of ophthalmia and other diseases of the eyes in these countries, gave medical men ample scope for the exercise of their art, and thus a degree of proficiency was acquired, which caused the Egyptian oculist to be considered the most fitting offering one sovereign could make to another. Dental maladies, on the other hand, were seldom met with in Egypt, and it is to this cause, no doubt, that we may attribute the general silence of authors respecting their proficiency as dentists. It is well known that a carious tooth is seldom to be met with in any of their ancient mummies. Herodotus, the first Greek historian, says, "The art of medicine is so practiced in Egypt, that there is found an individual healer for each individual distemper. Hence the whole country is filled with healers; some take charge of the disorder of the eyes, others of those of the head, others of those of the *teeth*, others of those of the belly, and others of secret diseases." From the hereditary character of these several offices, we may imagine that a high degree of proficiency would be acquired in the various specialties.

The ancients, we find, were generally opposed to the extraction of teeth, and advised various remedies instead, such as plugging with lead, (plumbing,) plugging with iron, galbanum, wax, the application of the actual cautery, the wearing of charms, &c. When the operation was deemed imperative, a serrated instrument, called by the Greeks *rizagra*, was first employed to shaken or loosen the tooth; the head of the patient was then placed between the knees of the operator, in order to keep it steady, and should the tooth not come out readily, a kind of hook or lever was inserted under it, on all sides, so as to raise it a little, before again attempting its extraction, when the operation was completed by pulling it perpendicularly. The best received authorities of the day recommend great hesitation and precaution before proceeding to the extraction of teeth.

Coelius condemns the inconsiderate extraction of teeth, and urges, above all things, "that care should be taken in the removal of those that are firmly fixed, as the adjacent parts, particularly the eyes, are always affected by the operation, and frequently it is not only one tooth, but the entire jaw that aches, in such a manner as to render it necessary to extract all the teeth, in order to obtain relief." Another states that when a tooth which is loose or painful is to be extracted, "the nose of the patient should be rubbed with brown sugar and ivy and green oil; he is advised to hold his breath, a stone is then placed between his teeth, and he is made to close his mouth; the fluid which causes the pain is then seen to flow from the mouth in such quantity as frequently to fill three pots; after having cleansed the nose with pure oil, and rinsed the mouth with wine, the tooth is no longer painful, and may be easily extracted." "To preserve one's self to a certainty from toothache, it is only necessary, at the return of the swallow in the spring of the year, to repair silently to the border of a clear rivulet, take some of the water in the mouth, and rub the tooth with the forefinger of both hands, and repeat a certain form of words. Another recommends that the roots be anointed with flour and the juice of tithymal, after which the teeth will fall out."

In pursuing the subject of extraction of teeth, allow me to offer a few remarks on the instruments employed for that purpose, both ancient and modern; note some of the most important improvements, and point out the style of instruments which, from long experience, I have been led to prefer, and, as far as practicable, show the mode of applying them to the teeth to be removed.

Among the first instruments of which we have any account, is the *vulsella*, described by Celsus, about the commencement of the Christian era ; it is said to have been a kind of forceps, the beaks resembling the bill of a parrot; this instrument, with slight alterations, came in time to be known as the hawksbill forceps, or *davier* of the French, and in connection with various levers and punches, continued in use for several centuries. The *elevator* next succeeded the forceps, and if we may judge from the description given of the method of using it, we shall be forced to the conviction that the operation of extracting teeth lost none of its terrors by the change. Following this, we have an instrument similar in its action to a key, furnished with claw and fulcrum, but lacking the power and safety of the latter. Among the levers employed, we find the *peid-de-biche* (hind's foot,) *langue-de-carp* (carp's tongue,) or *trevelin* of Lecluse, for the extraction of roots: it is a curved lever, terminating in a bayonet point, and was said to be a perfect instrument for the extraction of wisdom teeth, by placing it between it and the second molar. Another called the lever *a-crochet-et-a-plague* (the lever with hook and plate,) bears some resemblance to the key, though much inferior to that instrument. The *pelican*, an instrument formerly much in use, was furnished with a lever, fulcrum and claw, and approximated more closely to the key than any of its predecessors. In the early part of the 18th century, the *key* instrument, invented by Garengeot, came into use ; the facility of acquiring command of this instrument, and the amount of force it was capable of exerting, soon brought it into general favor, and it must be admitted that where but one instrument could be obtained, none would answer all purposes as well as the key ; its defects were sought to be remedied from time to time by the ingenuity of Van Butchell, Spence, Saviny, Duval and a host of others; to the last named gentleman we are indebted for the introduction of the movable fulcrum, one of the most valuable improvements which this instrument has undergone.

To Mr. Cartwright, of London, we are indebted for the first return to the use of the forceps; he so improved them as to enhance very much their efficiency, and about the year 1830 first brought them to public notice; they soon came to be regarded as safe and more reliable than the key. Mr. Snell, taking up the idea of Cartwright, soon followed in the line of improvements, by adapting the beaks to the neck of the different classes of teeth, by means of nibs, grooves and crescents, and added to the better application of the extractive force, by bending one of the handles so as to pass round the hand of the operator, thus preventing the liability of slipping. Dr. J. F. Flagg, of Boston, is entitled to the credit of an improvement in the hawksbill forceps, which consisted in more accurately adapting its form to the necks of the teeth, and at the same time giving a shape and position to the handles, which would enable the operator to apply the necessary power to greater advantage. A further improvement on Snell's instruments, by Prof. C. A. Harris, consisted in such a change of the handles, as brought the motive power, or hand, near on a line with the long axis of the tooth, while the change in the angles and curves enabled the operator to bring the hand nearer to his chest, and under the instrument, thus giving greater command over it, without any loss of power.

Dr. Edward P Church supplied a want long felt by the profession, in the invention of forceps forming two nearly right angles, above the joint, for the extraction of the superior wisdom teeth.

Dr. J. W. Crane, of New York, invented two pairs of forceps for the extraction of lower molars: one for the first and second, on either side, and the other for the wisdom teeth.

Dr. Hullihen's compound screw forceps, for the extraction of the roots of incisors and canine, in the upper jaw, you are all familiar with.

To Dr. E. Maynard, of Washington, D. C., we are indebted for forceps designed for the removal of the roots of upper molar teeth, before they have become separated from each other; there are two pairs, one for each side of the mouth, and though not often brought into requisition, they are admirably adapted to the class of cases for which they are intended.

Dr. Elliott has furnished an instrument for the extraction of the roots of molar and bicuspid teeth; but of its merits we are not prepared to speak.

The forceps bearing the name of Dr. Physick are found to be very useful in separating the roots of lower molars previous to their removal; they have been highly spoken of by some operators for the extraction of lower wisdom teeth.

John Tomes, of London, claims to have made valuable improvements in tooth forceps.

Mr. Fay and Mr. Matthews, both of England, introduced, some years ago, what was known as the *adjusted forceps;* they were brought to notice by both these gentlemen about the same time, neither being aware of the other's improvement. The advantages possessed by these instruments consist in their anatomical adaption to the neck and crowns of the several classes of teeth, and this we regard as a *sine qua non* in the fabrication of dental forceps.

The importance of starting out in the profession with good instruments cannot be over-estimated. With those first adopted we become most familiar from daily association, and while their defects may be numerous and radical, we are sometimes slow to recognize and correct them. I have met with instruments for extracting teeth, in the case of both physicians and dentists, that would set at defiance all the known laws of mechanics, and overmatch the ingenuity of the most skillful artisan to adapt them to any useful purpose. Our advice invariably in such cases is, throw them away, put them beyond your reach, and thus reduce the chances of fracture and other unpleasant complications. It is too frequently the case that young beginners procure such instruments as are pointed out to them, or take up those of some one retiring from practice, and seldom or never entertain the idea of improvement. We are happy to say, however, that this is not the rule; valuable improvements have been made from time to time, and the result of a combination of these are now to be found in the hands of a large portion of the operators of the present day. The most important requisites in forceps we conceive to be: 1st. A proper adaptation of the beaks to the necks and crowns of the teeth. 2d. The handles should be as near on a line with the longest axis of the tooth as possible, yet sufficiently bent to avoid coming in contact with the opposite jaw. 3d. The length of the handle should conform to the hand of the operator; long handles are particularly objectionable. 4th. The handles should be bent in such a manner as to place the operator in the most favorable position in relation to the patient. This is generally conceded to be on the right side, or right and rear. The head is best controlled in this position.

To insure success in the extraction of teeth, a careful diagnosis is of the first importance; an accurate acquaintance with the normal and pathological conditions of the teeth and their surroundings, and a sound judgment in relation to the obstacles to be encountered, is calculated to inspire the operator with a confidence in his own practical abilities, which will enable him to meet the physical indications in each individual case.

Another object to be aimed at is to gain the confidence of the patient. A kind and affable demeanor on the part of the operator, accompanied with candor and firmness, will seldom fail to accomplish this object. All unnecessary display of instruments should be avoided, and the surroundings of such a character as to allay the fearful apprehensions of the weak and timid. These conditions being complied with, we are now prepared to select the instruments best suited to the case in hand.

For the removal of the upper incisiors, the instrument most frequently employed has straight beaks, and on a line with the handle, both blades grooved and crescent-shaped at point; to conform to the necks of this class of teeth, the bowl or beaks should be sufficiently separated to admit the crown without pressure, as the object should be to grasp the tooth at or above the neck, in order to secure the best result. It will be found most convenient to have the lower handle bent, to pass round the little finger of the operator; the motion which I have found most effective in removing these teeth is the downward, or inward, after a slight rotary movement; the alveolus being shorter and thinner on the inner side, the attachments are more readily sundered by a motion in this direction. The instrument which I prefer for extracting the upper bicuspid differs from the one just described, in having the beaks narrower, and bent at an angle of about forty degrees, with straight handles; to give the necessary strength to this instrument, we should add to the thickness of the beak what it lacks in width. One instrument answers for both sides of the mouth. The rotary motion is inadmissible in the removal of these teeth, owing to conformation and position; the outward and inward motions should be given, particularly the latter. The value I set upon this instrument may be estimated when it is known that I use it for all roots in the upper jaw, after they have become separated from each other, and not unfrequently have extracted all the upper incisors, canine and bicuspids, with it alone.

The upper molar tooth having three roots, two buccal and one palatine, instruments for their extraction should be constructed to meet this anatomical arrangement; hence the necessity for two pairs, right and left, straight handles, and the beaks sufficiently wide to take in all the crown, and bent at an angle of about forty degrees; the point of the inner beak should be curved, so as to embrace closely the palatine root, while the outer one requires a double groove, terminating near the center in a strong, sharp nib. In arresting the tooth, this nib is forced into the depression between the buccal roots, when the inward and outward motion is given, and the organ dislodged. I prefer to have one of the handles curved, as in the incisor instrument.

The forceps previously noticed as the invention of Dr. Church are well calculated for the extraction of the upper wisdom teeth; the beaks are parallel with the handles, but removed about one inch from the same line by means of two angles above the joint; the points are both crescent-shaped. It is but seldom that we meet with a divergence in the roots of these teeth, hence the nib is not required. This instrument answers equally well for both sides of the mouth.

Passing to the lower jaw, we select an instrument for the removal of the incisors, canine and bicuspids, similar to the one employed for the upper bicuspids, the only difference being in the angle of the beaks, that of the upper being about forty degrees, while the one under consideration has an angle of sixty degrees at least; the handles straight, and beaks narrow but stout. With this instrument I remove the ten anterior teeth in the lower jaw, and all the roots of the lower molars after they have become separated from each other.

For the extraction of the lower molar teeth, one instrument is sufficient for both sides of the mouth. These teeth having two roots, both beaks of the forceps should be furnished with stout nibs, to penetrate the bifurcation; holding the instrument in the right hand, as if about to use it, and taking the shank, or joint, as the base, we have the handles bent toward the left hand of the operator at an angle of forty degrees, while the beaks present the same angle in a forward direction, one handle bent around the hand, and the bowl to conform with those previously described. This instrument I regard as decidedly the best I have ever met with for the class of teeth for which it is intended.

For the extraction of the inferior *dens sapientia*, an instrument with straight handles is to be preferred; the beaks slightly narrower than those last named, and bent at an angle of sixty degrees, both points crescent-shaped; the shank should be as small as is consistent with the required strength, in order to afford a full view of the parts to be operated upon ; as the roots of these teeth usually turn back in the ramus of the jaw, to a greater or less extent, it becomes necessary to have other appliances at hand, to meet the emergencies. The Physick forceps may occasionally be found useful here. I have not employed them to any considerable extent for this purpose. A stout lever, with angles similar to those of the upper wisdom forceps, will be found very valuable, introduced behind the second molar; the force should be applied to the wisdom tooth in an upward and backward directions, following every movement with the eye, in order to guard against forcing the tooth into the throat of the patient.

Having now described all the instruments necessary for the ordinary operations of extraction, let me call your attention to some of those employed for *extraordinary* cases; and first, for the removal of the upper incisor and canine roots, we have the conical screw, compound screw forceps, punch and gouge. I have tried them all, but for a number of years past, have depended almost entirely upon my upper root forceps. Where the crowns of upper molars have been broken off, or remain by caries, yet an unbroken connection remains between the roots, I have found the instrument invented by Dr. Maynard to excel all others that I have met with. The upper beak forms a sharp, strong hook, bent in such a manner as to be made to readily pass through the junction of the roots, while the lower beak is similar to those of the upper molar forceps; the motion is downward or inward, readily wrenching the organ from its attachments ; or when the union between the roots is too slight to resist the force, a separation takes place, when they are readily removed by the upper root instrument. Where the same condition exists in relation to the lower molars, I resort at once to the Physick forceps to separate the roots, and then remove them with the lower root forceps. It is sometimes necessary to extract teeth on account of their irregularity. Where three stand together in a group, it will be found that narrow, strong beaks are indispensable; the locality of the deviating organ will at once suggest to the mind of the operator the form of instrument best calculated to remove the difficulty.

I have not thought it necessary to occupy your time with any remarks in regard to the extraction of deciduous teeth. I have but one instrument specially devoted to this class; it is a small pair of forceps from the manufactory of Charriere, of Paris. They are the neatest, most convenient, and best adapted forceps I have ever met with, for the removal of deciduous molars; for all other teeth of the first set, I depend upon those already described.

A diversity of opinion exists in regard to the propriety of lancing the gums as preliminary to extraction. It is contended by some that the attachment of the gum to the neck of a tooth is always but slight and the extent inconsiderable, and that therefore its severance can have little or no influence in lessening the amount of force required in extraction. Again, it is admitted that there is a difference; that the gums should be separated from the teeth before removal; but for this purpose it is suggested that sharp forceps, driven forcibly into the gums, does the work more thoroughly, and is attended with no more pain than that produced by the lancet. Without stopping to combat these opinions, allow me to say, that I regard the lancet as one of the most important instruments in my case. I do not invariably employ it, but it is my practice to lance well, and I do contend that no timid hand should guide the blade. If the instrument is carefully kept, with a smooth and keen edge, the pain attending its use is inconsiderable, and more than counterbalanced by the increased facilities afforded for the expeditious and safe removal of the offending organ. The common, thin, flat handle is liable to turn in the hand; I have found an octagon handle less liable to do so, and much to be preferred.

And now a word in regard to the relative position of operator and patient. This is a matter concerning which no rules can be laid down. The form of instrument employed controls the operator in a great degree as to the position he assumes. With instruments of the hawksbill variety, the operator is compelled to follow the inclination of the instrument, sometimes on one side of the patient, and sometimes on the other; while with the instruments here described, the operator is never required to leave the right side of the patient, and is thus the better able to control the head with his left hand. The only deviation from this rule is in the extraction of the right inferior bicuspids and wisdom tooth, when he turns and faces the patient, though still retaining his position on the right side. Another important point is to have the operating chair neither too high nor too low. In a word the surroundings must be such as will best enable us to command the situation; for this purpose I have two small, separate platforms, each about four inches in height, placed one upon the other, at the back of my chair; the upper one is easily moved by the foot, and is always ready in tim^ of need. In extracting from the lower jaw, we get upon one or the other of these platforms, as the case may require; while, in operations in the upper jaw, we remain on the floor, or mount the first platform, if need be. To be able to distinguish promptly the requirements in each case that presents itself, includes not only a thorough knowledge of the buccal cavity in health and disease, but a familiarity with the various forms of instruments, and their modes of action. In this way alone can we acquire that manual dexterity which should ever distinguish the accomplished surgeon.

